# Supplementary material for: 3D deconvolution of human skin immune architecture with Multiplex Annotated Tissue Imaging System
Source: Sci Adv. 2023 Jun 7;9(23):eadf9491. doi: 10.1126/sciadv.adf9491 (PMC10246893; doi:10.1126/sciadv.adf9491)
Supplement: Supplementary file 1 — Figs. S1 to S6 Tables S1 to S4 Legend for movie S1 [file sciadv.adf9491_sm.pdf]

Science Advances
AAAS

Supplementary Materials for

3D deconvolution of human skin immune architecture with Multiplex Annotated Tissue Imaging System

Manon Scholaert *et al.*

Corresponding author: Nicolas Gaudenzio, nicolas.gaudenzio@inserm.fr

Sci. Adv. **9**, eadf9491 (2023)
DOI: 10.1126/sciadv.adf9491

The PDF file includes:

Figs. S1 to S6
Tables S1 to S4
Legend for movie S1

Other Supplementary Material for this manuscript includes the following:

Movie S1

Supplementary Figures and legends

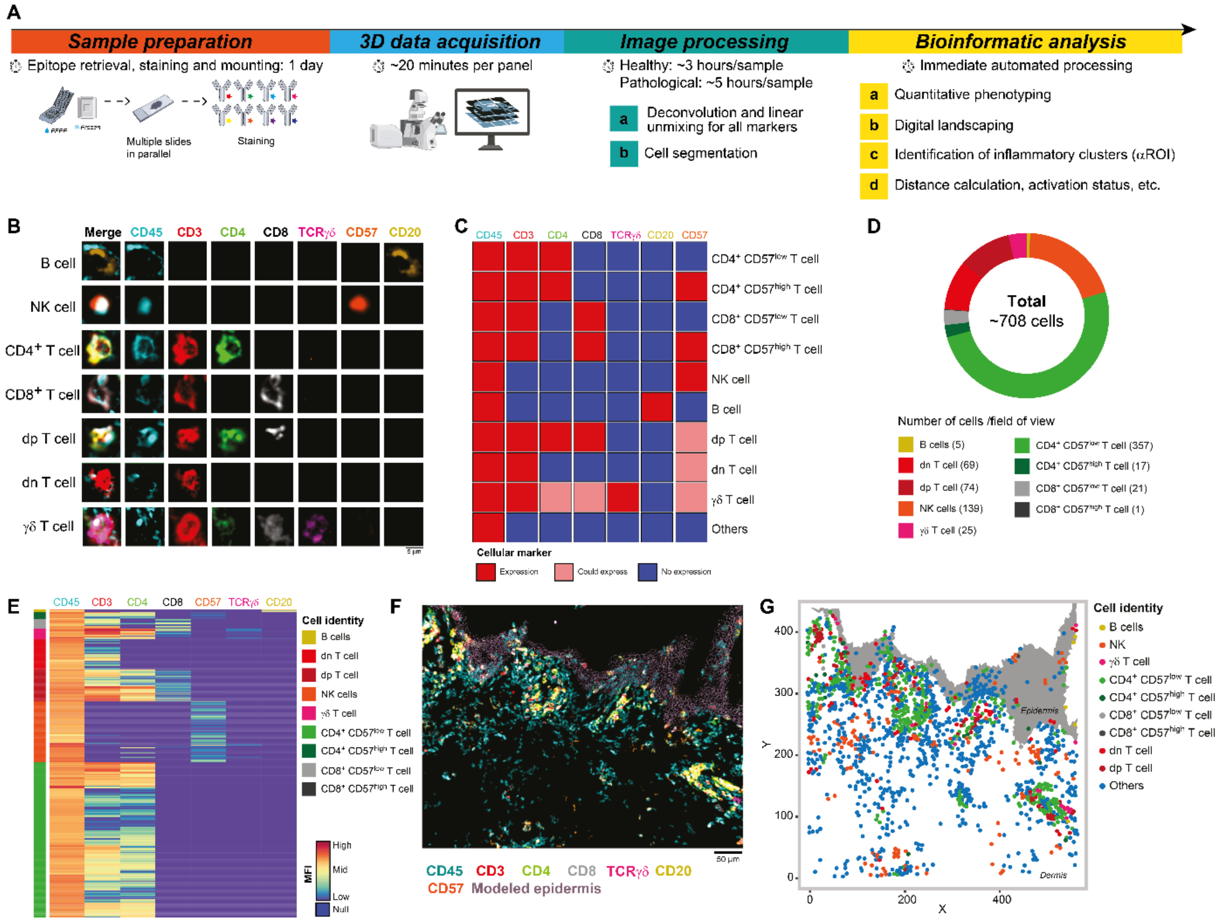

Figure S1. Main lymphoid cell populations annotated using MANTIS algorithm. A, Timeline of the MANTIS analytic pipeline. **B**, Examples of single-cell staining of all used biomarkers in identified lymphoid cells. Scale bar: 5 µm. **C**, MANTIS attribution matrix for the lymphoid panel. **D**, Tissue annotation and cell proportion of diseased skin (SLE patient). **E**, Heatmap of mean fluorescence intensity levels of used markers in identified lymphoid cells (colored scale). **F,G,** Representative 3-D confocal multiplex image (F) and associated digital map generated with MANTIS (G) of the lymphoid panel in diseased (SLE) skin. Scale bar: 50 µm.

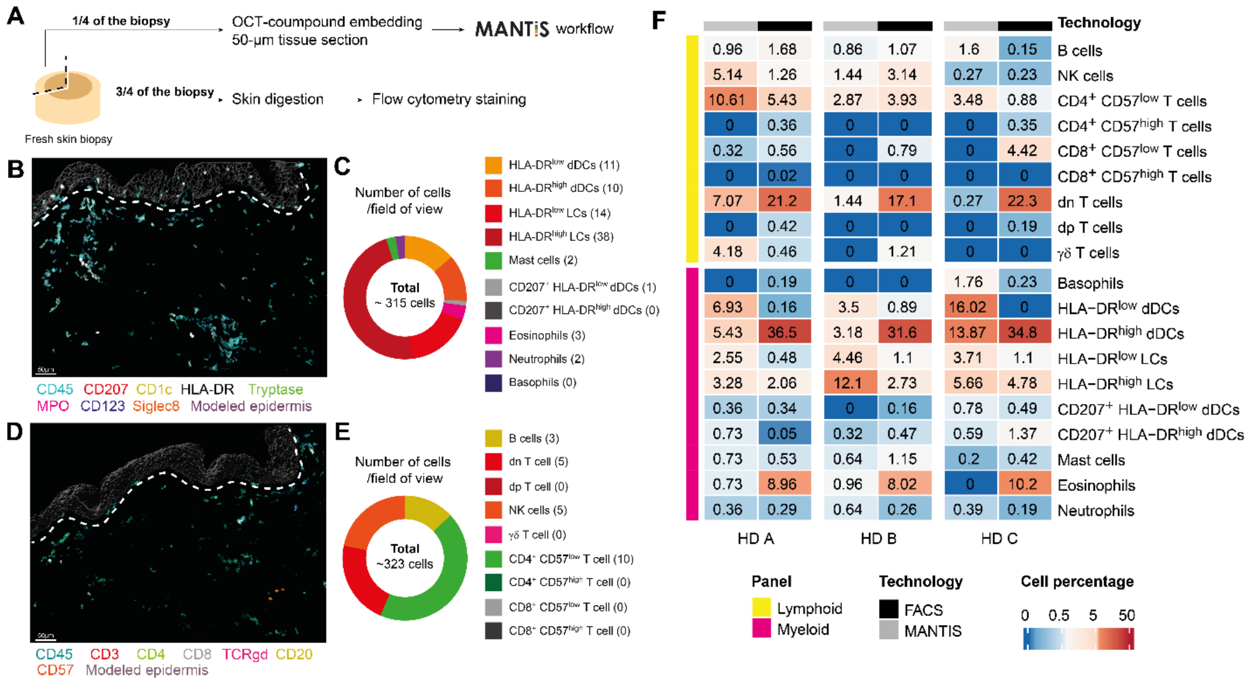

Cell type	HD A MANTIS	HD A FACS	HD B MANTIS	HD B FACS	HD C MANTIS	HD C FACS
B cells	0.96	1.68	0.86	1.07	1.6	0.15
NK cells	5.14	1.26	1.44	3.14	0.27	0.23
CD4+ CD57low T cells	10.61	5.43	2.87	3.93	3.48	0.88
CD4+ CD57high T cells	0	0.36	0	0	0	0.35
CD8+ CD57low T cells	0.32	0.56	0	0.79	0	4.42
CD8+ CD57high T cells	0	0.02	0	0	0	0
dn T cells	7.07	21.2	1.44	17.1	0.27	22.3
dp T cells	0	0.42	0	0	0	0.19
γδ T cells	4.18	0.46	0	1.21	0	0
Basophils	0	0.19	0	0	1.76	0.23
HLA-DRlow dDCs	6.93	0.16	3.5	0.89	16.02	0
HLA-DRhigh dDCs	5.43	36.5	3.18	31.6	13.87	34.8
HLA-DRlow LCs	2.55	0.48	4.46	1.1	3.71	1.1
HLA-DRhigh LCs	3.28	2.06	12.1	2.73	5.66	4.78
CD207+ HLA-DRlow dDCs	0.36	0.34	0	0.16	0.78	0.49
CD207+ HLA-DRhigh dDCs	0.73	0.05	0.32	0.47	0.59	1.37
Mast cells	0.73	0.53	0.64	1.15	0.2	0.42
Eosinophils	0.73	8.96	0.96	8.02	0	10.2
Neutrophils	0.36	0.29	0.64	0.26	0.39	0.19

Figure S2. MANTIS and flow cytometry methods show similar proportions of immune cells, except HLA-DR^{high} dDCs, eosinophils and double-negative T cells. A, MANTIS versus flow cytometry results comparison. A 23 mm-diameter human skin biopsy was cut into two pieces. One fourth of the biopsy was embedded in OCT, 50µm sections were generated and the slides were analyzed using the MANTIS pipelines previously described (myeloid and lymphoid panels). The three fourth remaining of the biopsy were digested, the resulting cell suspension was stained with the same lymphoid and myeloid panels and a flow cytometry analysis was performed. **B,C,** Representative 3-D confocal multiplex images of pre-designed MANTIS myeloid panel (B) and tissue annotation using MANTIS pipeline (C) of healthy skin. **D,E,** Representative 3-D confocal multiplex images of pre-designed MANTIS lymphoid panel (D) and tissue annotation using MANTIS pipeline (E) of healthy skin. **F**, Representative heatmap of lymphoid and myeloid cell proportions using MANTIS and flow cytometry methods on 3 different donors in logarithmic scale. Scale bar: 50 µm. *FACS: Fluorescence Activated Cell Sorting.*

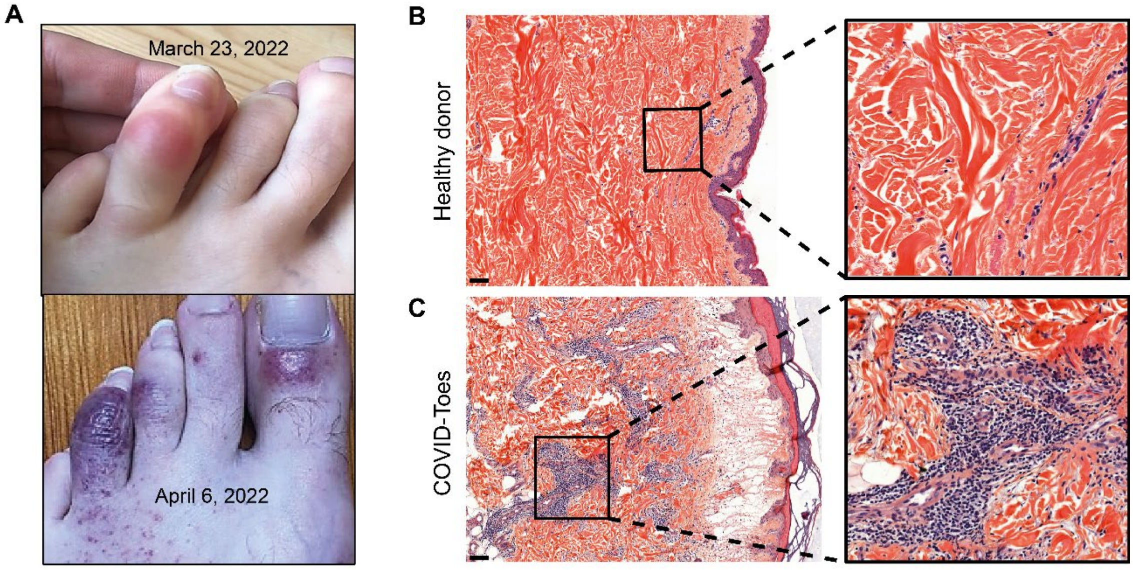

Figure S3. COVID-toes and associated histology. A, Evolution of a chilblain-like lesion in a COVID patient between March 2022 (upper panel) and April 2022 (lower panel). **B, C**, Comparison of H&E staining of a healthy-looking skin sample (B), and a chilblain-like lesion associated with COVID (COVID-T=toes), showing immune infiltrates (C). Scale bar: 100µm.

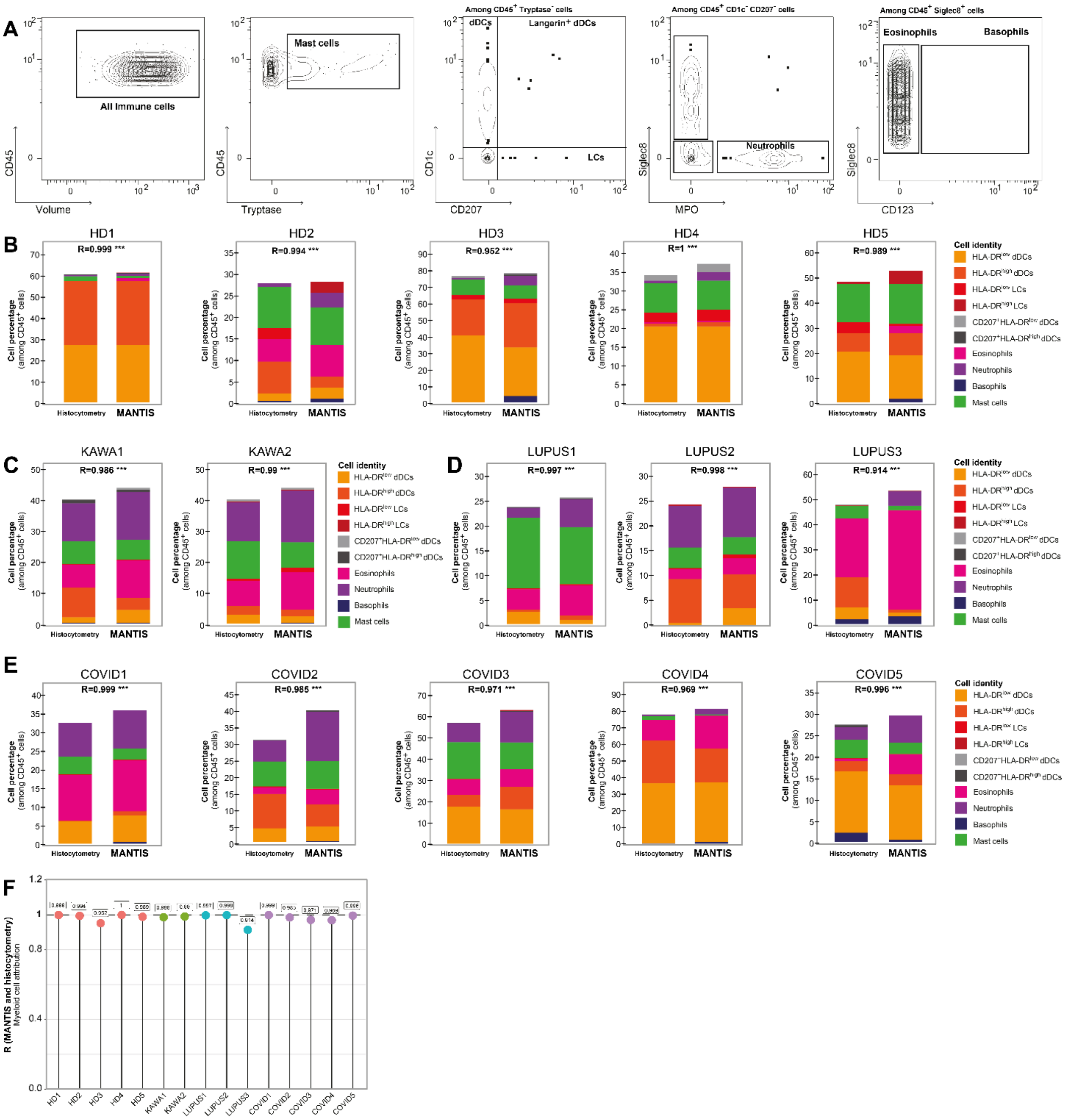

Figure S4. Quantitative validation of MANTIS myeloid tissue annotation with classical histo-cytometry. A, Gating strategies of identified myeloid cell populations using FlowJo. **B-E**, Comparison of cell type percentages using classical histo-cytometry or MANTIS and associated Pearson correlation coefficient in healthy skin (**B**) and Kawasaki (**C**), SLE (**D**) and COVID-toes (**E**) lesions. ***P<0.001, Pearson correlation test. **F**, Lollipop chart of Pearson correlation coefficients of all samples comparing histo-cytometry and MANTIS cell attribution.

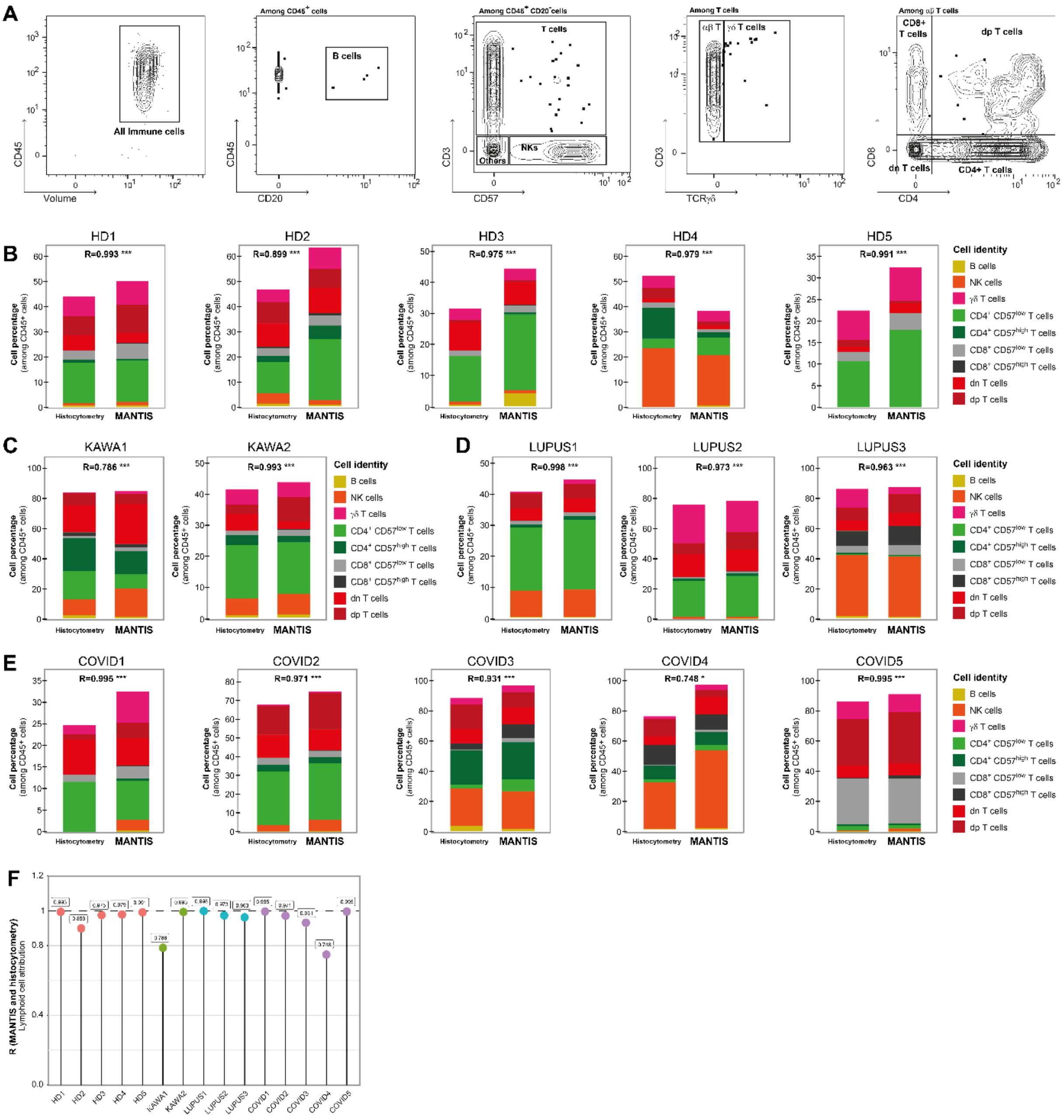

Figure S5. Quantitative validation of MANTIS lymphoid tissue annotation with classical histo-cytometry. A, Gating strategies of identified lymphoid cell populations using FlowJo. **B-E**, Comparison of cell type percentages using classical histo-cytometry or MANTIS and associated Pearson correlation coefficient in healthy skin (B) and Kawasaki (C), SLE (D) and COVID-toes (E) lesions. *P<0.05 ***P<0.001, Pearson correlation test. **F**, Lollipop chart of Pearson correlation coefficients of all samples comparing histo-cytometry and MANTIS cell attribution.

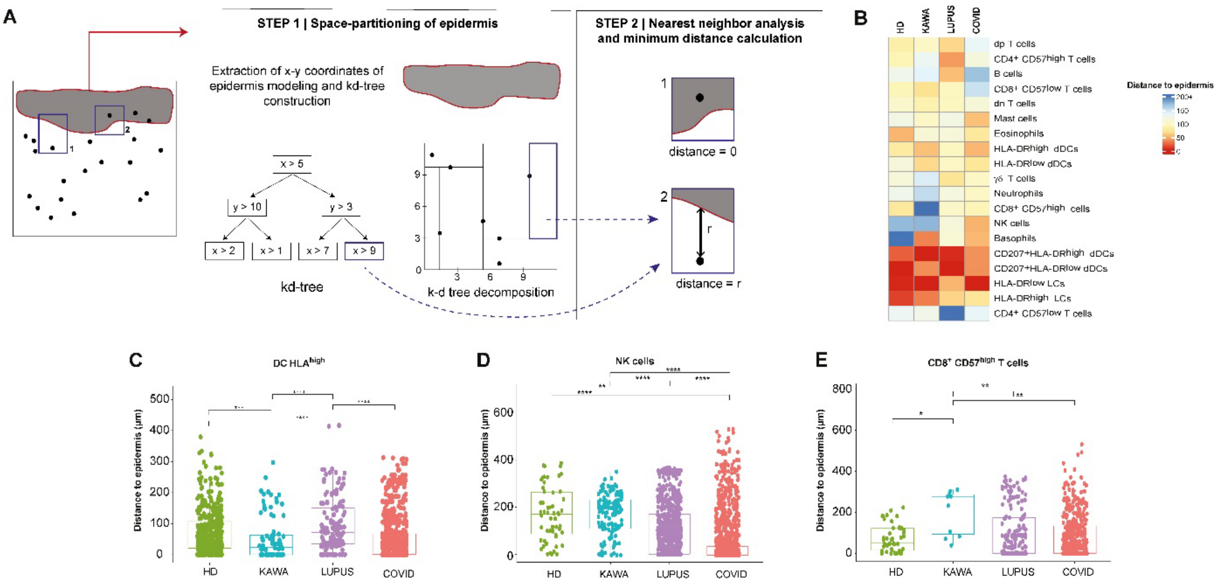

Figure S6. 3-D spatial distribution of immune cells and structural elements is computed using MANTIS. A, Quick nearest neighbor computation using KD-tree space-partitioning. *xy* coordinates of the epidermis were stored based on tree decision, using KD-Tree. Nearest neighbor search using KD-Tree point storage was then computed for each detected cell, and the minimal distance was calculated. **B**, Heatmap of mean distance to epidermis (colored scale) per cell type in healthy and pathological skin. **C-E**, Mean distance to epidermis (in µm) of dDC HLA^{high} (C), NK cells (D) and CD8^{+} CD57^{high} T cells (E) in healthy and pathological skin. Mean ± SEM; *P<0.05, **P<0.01, ***P<0.001 One-way ANOVA.

Supplementary Tables and legends

Antibody	Clone	Fluorochrome	Concent.	Reference
CD45	HI30	AF-532	0.12 µg	Thermofisher, #58-0459-42
**Lymphoid panel**				
CD4	N1UG0	Unconjugated<br>*Revealed with anti-mouse AF488*	10 µg/ml	eBioscience™, #14-2444-82
CD3	Polyclonal	Unconjugated<br>*Revealed with anti-rabbit AF594*	10 µg/ml	Agilent, #A045229-2
CD20	2H7	APC-Cy7	20 µg/ml	Biolegend, #302313
TCR-γδ	B1	BV650	15 µg/ml	BD, #564156
CD8a	AMC908	eFluor 660	10 µg/ml	Thermofisher, #50-0008-80
CD57	TB01	eFluor 450	10 µg/ml	Thermofisher, #48-0577-41
**Myeloid panel**				
Siglec8	Polyclonal	Unconjugated<br>*Revealed with anti-rabbit AF405*	1:50	Thermofisher, #PA5-110774
CD1c	L161	Zenon AF488 Using Zenon Mouse IgG1 labeling kit (Thermofisher, #Z25000)	10 µg/ml	Biolegend, #331502
Tryptase	AA1	Zenon AF647<br>Using Zenon Mouse IgG1 labeling kit (Thermofisher, #Z25000)	1:50000	Abcam, #ab2378
CD207	923B7	AF546	5µg/ml	Biotechne, #DDX0373A546
MPO	Polyclonal	Unconjugated<br>*Revealed with anti-goat-AF594*	5 µg/ml	Biotechne, #AF3667
HLA	L243	AF700	10 µg/ml	Biotechne, #NB100-77855AF700
CD123	6H6	SB645	1 µg	eBioscience™, #64-1239-42

Table S1. Key resources. Classical and available unconjugated and conjugated antibodies were used in this study at indicated concentrations.

Antibody	Fluorochrome	Excitation laser (nm)	Laser power (%)	Detector type	Detection window
CD45	AF-532	532	6	HyD	535-578 nm
**Lymphoid panel**					
CD4	AF488	488	2.7	HyD	503-539 nm
CD3	AF594	552	6	HyD	603-633 nm
CD20	APC-Cy7	635	50	PMT	740-790 nm
TCR-gd	BV650	405	10	HyD	624-682 nm
CD8a	eFluor 660	635	67.2	HyD	651-693 nm
CD57	eFluor 450	405	10	HyD	415-479 nm
**Myeloid panel**					
Siglec8	AF405	405	3.67	HyD	409-470 nm
CD1c	AF488	488	1	HyD	505-536 nm
Tryptase	AF647	635	89.5	HyD	648-689 nm
CD207	AF546	552	4	HyD	558-588 nm
MPO	AF594	552	2.04	HyD	608-633 nm
HLA	AF700	635	50	PMT	699-782 nm
CD123	SB645	405	3.97	HyD	625-681 nm

Table S2. Microscope configuration of MANTIS acquisitions. Between-stack acquisition parameters were configurated using all available four lasers in visible range wavelengths and detectors (Hybrid [HyD] or photomultiplier [PMT]).

Table S3. All datasets corresponding to the myeloid panel. Single cell databases (.csv file) of all healthy samples and patients used to generate the presented data are available in the non-profit repository Dryad (doi:10.5061/dryad.rxwdbrvdm).

Table S4. All datasets corresponding to the lymphoid panel. Single cell databases (.csv file) of all healthy samples and patients used to generate the presented data are available in the non-profit repository Dryad (doi:10.5061/dryad.rxwdbrvdm).

Video S1. Example of MANTIS user interface. This video shows the gating possibilities on the software interface and associated single cell quantitative analyzes of biomarkers.
